# An investigation of the use of ethyl chloride and meloxicam to decrease the pain associated with a single or double incision method of castration in piglets

**DOI:** 10.3389/fpain.2023.1113039

**Published:** 2023-07-28

**Authors:** Arlene Garcia, Mhairi Sutherland, Gizell Vasquez, Adrian Quintana, Garrett Thompson, Jemma Willis, Shelbie Chandler, Kiran Niure, John McGlone

**Affiliations:** ^1^ School of Veterinary Medicine, Texas Tech University, Amarillo, TX, United States; ^2^ Ruaura Research Centre, AgResearch Ltd., Hamilton, New Zealand; ^3^ Animal and Food Sciences Department, Texas Tech University, Lubbock, TX, United States

**Keywords:** pigs, castration, behavior, physiology, performance, vapocoolant, pain

## Abstract

Castration is a stressful and painful procedure that can impact swine welfare negatively. The objectives of this study were to (1) evaluate the effect of one incision compared to two incisions and the use of a topical vapocoolant (VAPO; ethyl chloride; a topical anesthetic) applied before castration and (2) evaluate the most effective combination in reducing pain in objective 1 and the use of Metacam®; meloxicam before castration on measures of performance, behavior, and physiology. Study 1 consisted of six treatment groups (*N* = 27 pigs per treatment) and included: nothing (NO); sham castrated (SH); one incision castration (C1); one incision castration plus VAPO (C1V); two incision castration (C2); two incision castration plus VAPO (C2V). Body weights and blood samples were taken at baseline and other time points after castration. Behavior measures were collected for 24 h after castration. Wound scores were collected daily for 10 days. The C1 pigs and C1V pigs were significantly heavier than the other castrated treatment groups but not different from NO and SH pigs. Vocalizations were louder for C1 and C1V pigs (*P* = 0.0015). Study 2 (*N* = 40 pigs per treatment) included: nothing (NO); one incision castration (C1); and one incision castration plus meloxicam administered 15 min before castration (C1M). The same measures (performance, behavior, and physiology) were collected as in Study 1. Performance measures and behavior did not differ among treatment groups. Physiological measures were only different for red blood cells (RBC; *P* = 0.0304). Pigs in C1 and C1M treatment groups had cortisol concentrations that were greater than the NO treatment group at 15 min post-castration (*P* < 0.05). The data collected give insight into the benefits of one-incision castration compared to 2-incision castration. However, the data only support a lower-level relief from acute pain associated with castration, as it is evident that pigs still experience stress at 15 min post-castration with or without the use of meloxicam. Further research could potentially identify the correct timing, route and dose for the administration of meloxicam.

## 1. Introduction

Physical castration is a common procedure performed on young piglets usually at 2–3 days of age and is a standard practice in many countries, including the United States. Castration is a procedure that causes prolonged pain and is detrimental to the welfare of piglets (1, 2). Therefore, some countries (such as the EU and Israel) are incorporating analgesia during castration and other mutilations (3), or are considering the use of long-acting analgesics or anesthetics in all castrated pigs (4, 5).

Pigs are castrated early in life to prevent boar taint in the meat of sexually mature males and to reduce aggressive behavior. During castration one incision can be made or two incisions, one on each side of the scrotum (1). The testicles are externalized, grasped, and pulled out, tearing the spermatic cord, or the spermatic cord is cut with the scalpel blade (6). Literature is limited on the benefits of one incision versus two incisions, but there is speculation that one incision would be less painful than two, considering that the skin of the scrotum and tissues have sensory and motor innervations (1). Additionally, there is anecdotal evidence to suggest wounds heal quicker when only one incision is made because the amount of tissue damage is less, so the pain would be less. However, there is no scientific evidence that suggests which method may be preferable from an animal welfare perspective.

Castration is usually performed without any type of analgesia (7–9) and therefore, it is a painful (8, 10, 11) and stressful procedure in young pigs. Various studies have shown that physical castration leads to changes in behavior, which may be indicative of pain (12–16). Additionally, physical castration can lead to acute changes in physiology including the activation of the hypothalamic-pituitary-adrenal axis (HPA) and the activation of the sympathetic nervous system (SNS) (6, 17, 18). Physical castration in piglets also induces strong vocal responses (17–20). Screams (classified as having a higher frequency than squeal) have been reported as being more predominant during castration without anesthesia (19). These high-frequency calls are indicative of pain, mainly associated with the pulling and severing of the spermatic cord. Behavioral observations over a period of 5 days showed that physically castrated piglets spent significantly less time at the udder, had decreased activity while awake during the first 2.5 h, and overall tended to walk more throughout the 5 days post castration (6). Behaviors such as stiffness, prostration, and trembling are common for the first few hours after physical castration but in the days following, piglets show other pain-like behaviors like scratching their rump and tail wagging (6).

The use of general anesthetics and local anesthetics may be limited by the regulations and economics of the swine industry. Currently, the most used local anesthetic is lidocaine (21, 22). However, lidocaine injected into the scrotum and testicles can also cause additional pain and distress to piglets. Furthermore, lidocaine concentration is highest 3 min after injection, and thus, piglets would have to be castrated within that period, which may be impractical at farms because of the increased time and stress caused by repeatedly handling pigs to administer the injection and then castrate them (18). Ethyl chloride sprays are topical anesthetics with immediate skin anesthesia by rapid evaporation of volatile liquid, which produces a drop in skin temperature, resulting in temporary and swift interruption of skin sensation (23). Ethyl chloride has previously been used for breast needle biopsies, during intravenous cannulation (24, 25), and before immunizations (26–28) in humans. In livestock, they have been used to reduce the pain of ear tagging and ear notching in unweaned calves (29). Although the use of ethyl chloride in humans is widely reported to reduce pain prior to painful procedures, its use in farm/livestock animals is vaguely reported.

Meloxicam is a non-steroidal anti-inflammatory drug (NSAID) that has analgesic effects due to its peripheral anti-inflammatory actions (30). Meloxicam has been researched extensively for its postoperative analgesic effects in multiple species, including humans and pigs. Meloxicam has marketing authorization for use in non-infectious locomotor disorders to reduce symptoms of lameness and inflammation, but more recently for relief of postoperative pain associated with castration in piglets (meloxicam, 5 mg ml^−1^ solution for injection (3, 30). In a study, von Borell et al. (2) reported that 2 mg/kg BW meloxicam given intramuscularly 15 min before castration prevented a rise in cortisol concentration after castration, suggesting that it was effective in reducing pain. Therefore, one incision castration with the use of ethyl chloride in combination with meloxicam (0.4 mg/kg BW, according to the manufacturer's label) could reduce some of the pain associated with castration compared to not using anything at all. Therefore, this study was divided into two parts, Study 1 and Study 2. The objectives of study 1 were to evaluate the effect of one incision compared to two incisions for physical castration and the use of ethyl chloride applied before castration, and the objective for study 2 was to evaluate the effect of the best method found in objective 1 and the use of meloxicam before castration using measures of behavior, performance, and physiology.

## 2. Materials and methods

All animal procedures were approved by the Texas Tech University (TTU) Animal Care and Use Committee (protocol number: 17079-09). The experiment was conducted partially at the Texas Tech University New Deal Swine Facility and a commercial site.

Piglets were housed in farrowing crates with their dam. The farrowing crates were slatted, and piglets were on a milk-liquid diet. Heat lamps were provided in every crate. The temperature in the barns was kept at an average of 75°F.

### 2.1. Study 1

All male piglets were identified after farrowing and weighed the day prior to the study to assign them to one of six treatment groups (*N* = 27 pigs per treatment). Treatment groups consisted of control control/no surgical, NO; sham castrated, SH; one incision castration ± ethyl chloride, C1 or C1V; two incision castration ± ethyl chloride (VAPO), C2 or C2V. The NO pigs did not undergo any surgical procedures (but were handled and blood sampled in the same way as the other treatments). SH pigs had blood collected, underwent simulated castration (with a touch on the scrotum with a scalpel handle), and had a stream of cooled saline applied to simulate a stream of vapocoolant.

On the day of the study, pigs (3 days of age) were removed from their farrowing crate, weighed, and marked with livestock chalk according to their treatment group (also identifiable by ear notch). Randomly selected focal pigs (15 pigs/treatment group) were placed in a V-shaped trough where a baseline blood sample was taken. Pigs in the C1V and C2V groups were sprayed with ethyl chloride 10 cm away from each scrotum for 3 s and given 10 s for it to take effect. The ethyl chloride was then wiped off the skin with alcohol and the castration procedure was performed within 10–12 s of application (for two incisions spraying the open incision was avoided by holding a hand over the incision while holding the opposite testicle and spraying). All the handlers wore gloves for all the procedures and were trained with over 200 pigs to perform the castration procedures. Pigs were handled once if in the NO treatment for identification, weighing, and blood sampling. All other treatment groups were handled twice for identification, weighing, and blood sampling and then a second time for application of VAPO or Sham and castration. Castration (treatments C1, C2, C1V, and C2V) was conducted by placing the pig upside down between the handler's legs, exposing the anogenital region, and making either one or two vertical incisions on the scrotum to extract the testicle/s. Once the testicles were extracted and cut. Pigs were ear notched, and tail docked (as requested by the facility), and an antiseptic (iodine spray) was topically administered to the incision site/s and tail. Piglets were then returned to the farrowing crate with the sow.

#### 2.1.1. Performance measures

Pigs were weighed by placing the piglets in a tub on top of a digital scale. The scale was calibrated before weight collection by placing a stainless steel 2 kg weight on the scale to ensure the weight was accurate. The accuracy of the scale was 0.02 kg and weight data were recorded to the nearest hundredth of a kg. Weights were collected prior to beginning any procedures (baseline), 24, 48, and 72 h (because 24 h may only represent gut fill) post-treatment and at weaning (14 days). Weight change was calculated to determine the effect of the treatments.

#### 2.1.2. Behavior measures

Pig vocalizations during castration were analyzed using STREMODO (Stress-Scream Monitor and Documentation Unit), which were used for the analysis of vocalizations during castration (31, 32). After castration, piglet behaviors were observed every 10 min for 1 min for 1 h using handheld video recorders (Panasonic HC-V785K, Panasonic Corporation, Earth City, MO). After the first hour, the behavior was recorded via 10-min scan samples for 24 h using digital video recorders (DVR; Night Owl 1080p HD, Night Owl SP LLC, Naples, FL). Six trained and validated observers recorded walking, sitting, nursing, fighting, tail wagging, rump scratching, chomping, thrashing and other pain-like behaviors as described by Sutherland et al., 2012 and all observers were blind to the treatments.

#### 2.1.3. Physiological measures

Physiological measures were collected to determine if pigs might be immunosuppressed or if metabolic indicators show signs of tissue or systemic stress. Blood samples (2 ml) were collected via jugular venipuncture from randomly selected focal pigs (15 pigs/treatment group). Blood collection was performed prior to any procedures (baseline), and at the time in which cortisol is reported to peak (1), at 15, 30, and 180 min and 24 h. Blood was examined for hematological and blood chemistry changes that could occur during acute stress, including white blood cell (WBC) counts, differential counts for WBC populations, neutrophil: lymphocyte ratio [NL increases during stress (33)], hematocrit (HCT), red blood cells RBC), hemoglobin (HGB), mean corpuscular volume (MCV), mean corpuscular hemoglobin concentration (MCHC), red blood cell distribution width (RDW), platelets (PLT), mean platelet volume (MPV), platelet distribution width (PDW), glucose (GLU), total protein (TP), alkaline phosphatase (ALP), alanine aminotransferase (ALT), blood urea nitrogen (BUN), and creatinine (CRE).

#### 2.1.4. Wound healing

Wounds were assessed to determine possible detrimental effects due to castration or analgesic treatment according to Sutherland et al. (18). Pigs were numbered with colored livestock chalk to be able to identify them through video recordings. This also aided in preventing any bias while assessing wound scores/bruising. Pictures were taken and wounds were scored later by blinded accessors with only the wound visible to the observer.

Wounds after castration were scored daily for incision healing, using a 1–6 scale (1 = completely healed with no scab and 6 = fresh blood still present at the wound) and bruising, using a scale of 1–4 (developed by this group; where 1 = one-fourth of the scrotal area bruised, 2 = half of the scrotal area bruised, 3 = three-fourths of the scrotal area bruised, 4 = the entire scrotal area bruised. Wounds and bruising were recorded daily over a period of 10 days.

### 2.2. Study 2

Based on the findings from Study 1, all male pigs (3 days of age) were identified and weighed the day prior to beginning the study. Pigs (*N* = 40 pigs per treatment) were assigned to one of three treatment groups (no surgical incision, NO; one incision castration, C1; one incision castration plus an injection of meloxicam, C1M). On the day of the study, pigs were removed from their farrowing crate, weighed, and marked with livestock chalk according to their treatment group (also identifiable by ear notch) and randomly selected focal pigs (20 pigs/treatment group) were placed in a V-shaped trough where a baseline blood sample was taken. Pigs in the C1M group were injected with Metacam®; meloxicam (20 mg/ml; 0.4 mg/kg BW; depending on the treatment group) intramuscularly behind the ear in the order in which they were picked up (15 min prior to castration; and castrated in the same order they were first injected to castrate in the order of time lapse since injection). All pigs were handled twice for identification, weighing, blood sampling, intramuscular injection (only if in the C1M group), and then a second time for castration. Castration was conducted by placing the pig upside down between the handler's legs, exposing the anogenital region, and making one vertical incision on the scrotum to extract the testicles. Once the testicles were extracted, pigs were each notched and tail docked, and an antiseptic (iodine spray) was topically administered to the incision site and tail. Piglets were then returned to the farrowing crate with the sow. Methods carried out for taking the performance, behavioral, physiological, and wound healing measures in study 1 were replicated for study 2.

### 2.3. Data analyses

Data were found to adhere to the assumptions of the analysis of variance. Therefore, general linear mixed model analyses were performed using SAS for academics (2022, www.sas.com). The sow-litter-crate was the experimental unit. Each treatment was represented in each litter; therefore, the sow was treated as a random block effect and then treatments were evaluated. Blood samples were collected over time resulting in a repeated measures design. Wound scores were analyzed using chi-square.

#### 2.3.1. Study 1

The study was a randomized complete block design with six treatments and piglets' blood was sampled over five-time points. Thus, for blood measures, the data were RCB split plots over time. For measures of vocalizations and piglet performance, the analyses were simple RCB with six treatments. The model included effects of treatment, litter (random effect), litter by treatment interaction, time, and the time by treatment interaction. The litter by treatment interaction was the error term to test treatment effects; the residual error was used to test time and treatment by time effects. Each treatment had 27 pigs, with each treatment represented within each litter, for a total of 162 pigs. Least squares means were evaluated using the predicted difference test when a significant treatment effect (*P* < 0.05) was observed.

#### 2.3.2. Study 2

The study design was identical to that used in Study 1, except only three treatments were evaluated. Each treatment had 40 pigs from each litter, for a total of 120 pigs. For all measures, the main fixed effects were treatment, time, and crate. The interaction between treatment and time was included in the model with litter included as a random effect.

## 3. Results

### 3.1. Study 1

#### 3.1.1. Performance

**Figure 1:**
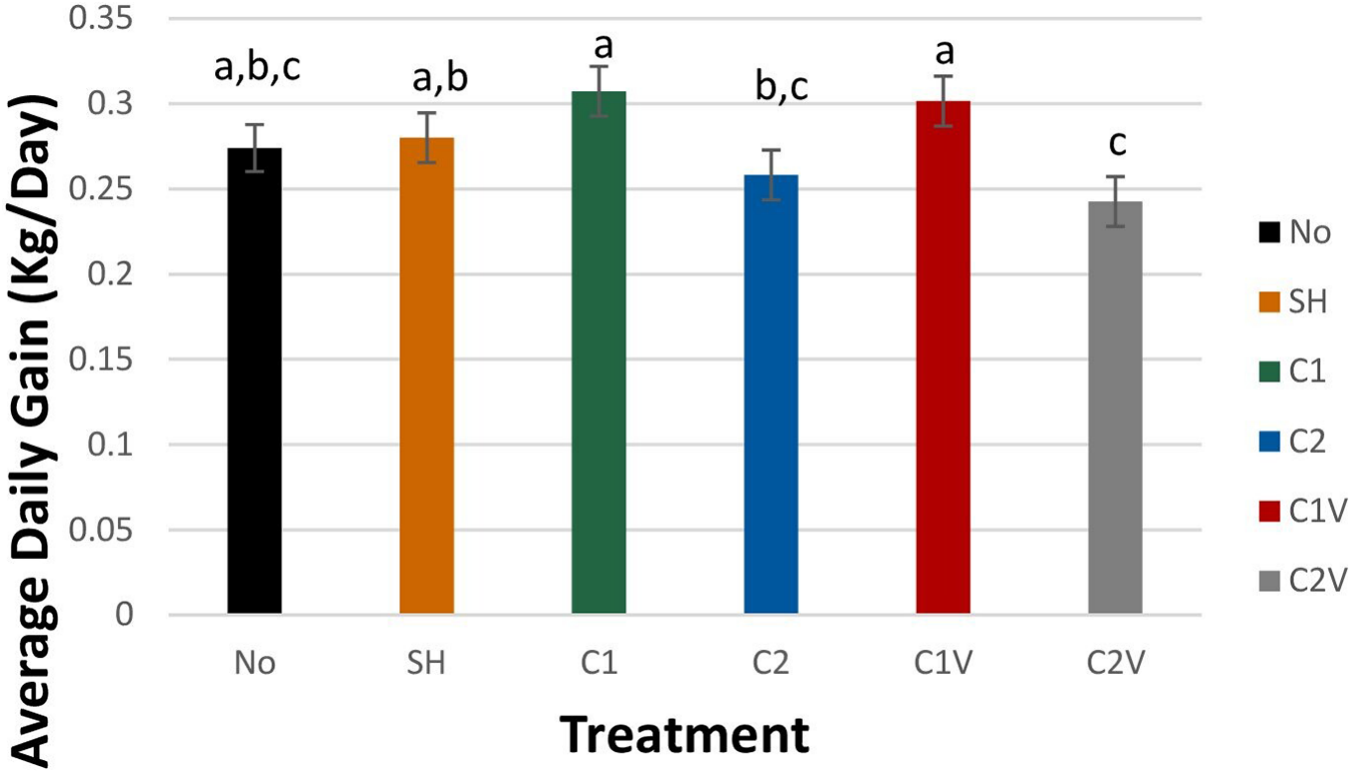
Study 1: LS means ± SEM for average daily gain of pigs castrated at 3 days of age using two methods of castration (treatments: 1 incision, C1; 2 incisions, C2; or simulated castration, SH; or not castrated, NO) with or without the use of a vapocoolant (V). *N* = 27 pigs per treatment group. ^a,b,c^Means with different superscripts differ at *P* < 0.05.

The castration method and the use of ethyl chloride had a significant effect on the average daily (ADG) (Figure 1). Pigs castrated with one incision (C1) and one incision plus ethyl chloride (C1V had significantly higher ADG (0.31 ± 0.02 and 0.30 ± 0.02 kg/day, respectively) than C2 and C2V castration groups (0.26 ± 0.02 and 0.24 ± 0.02 kg/day respectively). Pigs in C1 and C1V did not significantly differ in ADG from NO and SHAM pigs (0.27 ± 0.01 and 0.28 ± 0.01 kg/day, respectively).

#### 3.1.2. Behavior

**Figure 2:**
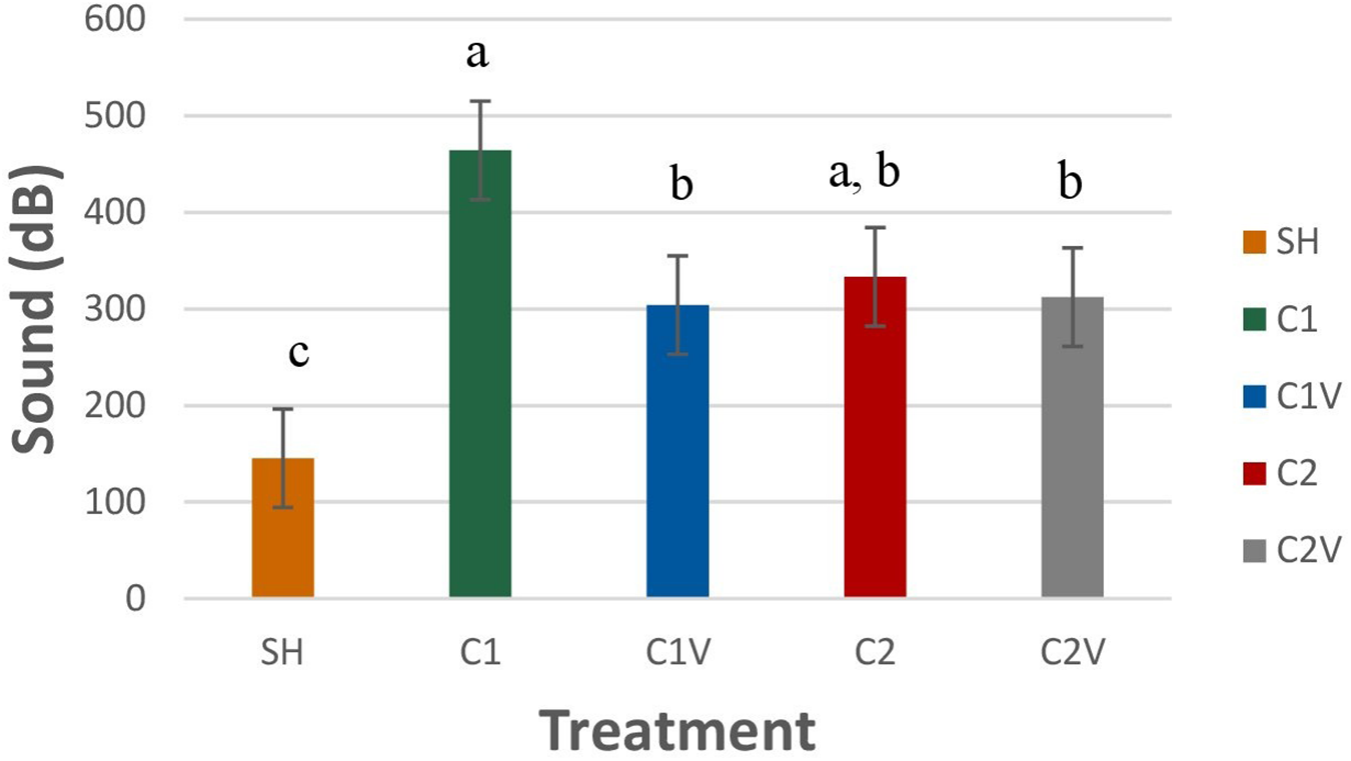
LS means ± SEM of the vocalizations of pigs castrated at 3 days of age using two methods of castration (treatments: 1 incision without vapocoolant, C1; 1 incision with vapocoolant, C1V; 2 incisions without vapocoolant, C2; 2 incisions with vapocoolant, C2V, simulated castration, SH; not castrated, NO). *N* = 27 pigs per treatment group. ^a,b,c^Means with different superscripts differ at *P* < 0.05.

Pig behaviors did not significantly differ among pigs, regardless of the treatment. Stress vocalizations recorded during castration were significantly different among treatment groups (Figure 2: *P* = 0.0015). Pigs in the C1 treatment group (464.20 ± 51.0 dB) had louder stress vocalizations than all the other treatment pigs but did not differ from C1V (333.21 ± 51.0 dB). C1V did not differ from C2 (304 ± 51.0 dB) and C2V (312.21 ± 51.0 dB).

#### 3.1.3. Hematology and blood chemistry

##### 3.1.3.1. Mean corpuscular volume

**Figure 3:**
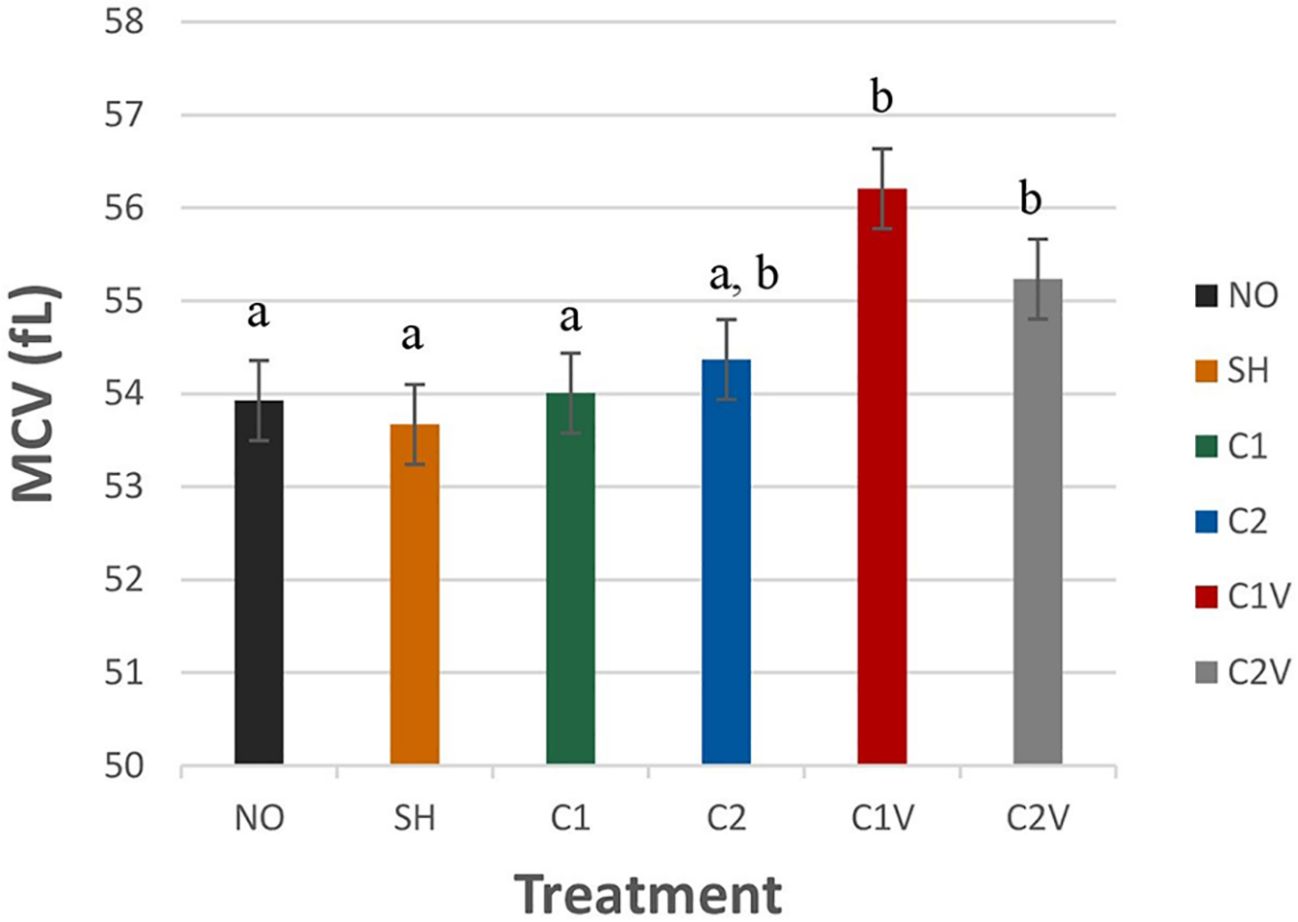
LS means ± SEM for mean corpuscular volume (MCV) of pigs castrated at 3 days of age using two methods of castration (1 incision, C1; 2 incision, C2; or simulated castration, SH; or not castrated, NO) with or without the use of a vapocoolant (V) (*P* = 0.0002). *N* = 27 pigs per treatment group. ^a,b^Means with different superscripts differ at *P* < 0.05.

Mean corpuscular volume (MCV) was significantly different among the treatment group (Figure 3; *P* = 0.0002). MCV levels were higher in C1V (56.02 ± 0.43 fl) and C2V pigs (55.23 ± 0.43 fl) compared to other pigs (*P* < 0.05). However, C2 pigs (54.36 ± 0.43 fl) were not significantly different that the C1V (56.02 ± 0.43 fl) or C2V (55.23 ± 0.43 fl) pigs nor other treatment groups.

##### 3.1.3.2. Mean corpuscular hemoglobin

**Figure 4:**
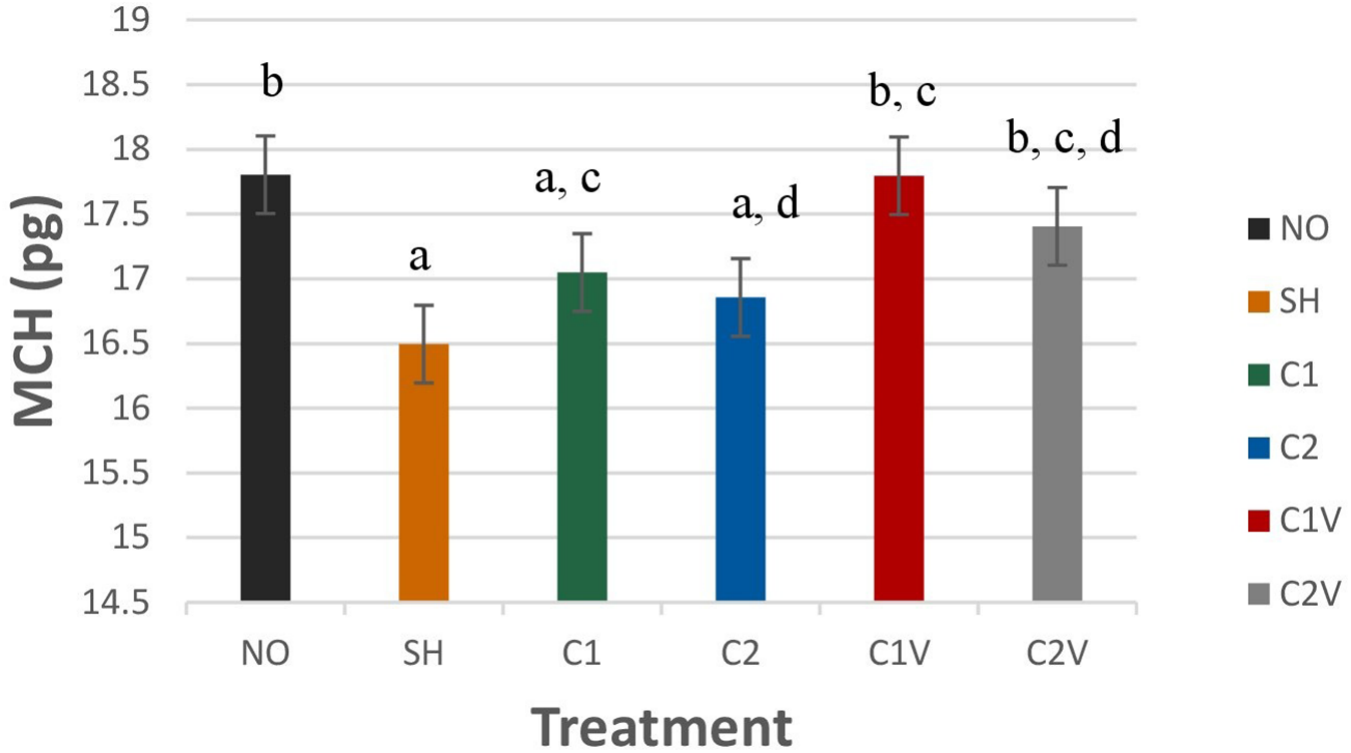
Study 1: LS means ± SEM for mean corpuscular hemoglobin (MCH) of pigs castrated at 3 days of age using two methods of castration (1 incision, C1; 2 incision, C2; or simulated castration, SH; or not castrated, NO) with or without the use of a vapocoolant (V) (*P* = 0.0053). *N* = 27 pigs per treatment group. ^a,b,c,d^Means with different superscripts differ at *P* < 0.05.

Mean corpuscular hemoglobin (MCH) was significantly different among treatment groups (Figure 4; *P* = 0.0053). SH pigs had the lowest values (16.49 ± 0.30 pg), but they were not different from C1 and C2 pigs (17.05 ± 0.30 pg and 16.86 ± 0.30 pg respectively).

##### 3.1.3.3. Mean corpuscular hemoglobin concentration

**Figure 5:**
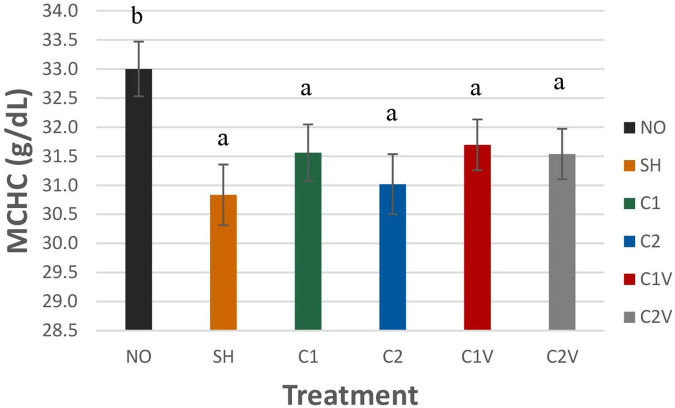
Study 1: LS means ± SEM for mean corpuscular hemoglobin concentration (MCHC) of pigs castrated at 3 days of age using two methods of castration (1 incision, C1; 2 incision, C2; or simulated castration, SH; or not castrated, NO) with or without the use of a vapocoolant (V). *N* = 27 pigs per treatment group. ^a,b^Means with different superscripts differ at *P* < 0.05.

Mean corpuscular hemoglobin concentration (MCHC) was significantly higher in NO pigs (33 ± 0.47 g/dl) compared to all other treatment groups (Figure 5; *P* = 0.0065).

##### 3.1.3.4. Red cell distribution width

**Figure 6:**
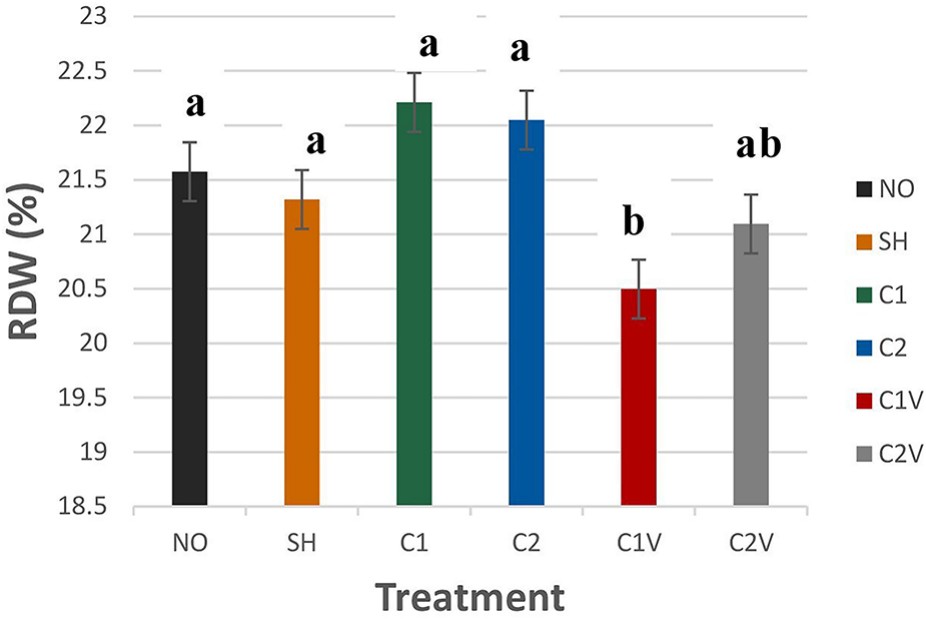
Study 1: LS means ± SEM for red cell distribution width (RDW) of pigs castrated at 3 days of age using two methods of castration (1 incision, C1; 2 incision, C2; or simulated castration, SH; or not castrated, NO) with or without the use of a vapocoolant (V) (*P* = 0.0001). *N* = 27 pigs per treatment group.

Red cell distribution width (RDW) was significantly different among treatment groups (Figure 6; *P* = 0.0001). C1V pigs (20.50 ± 0.27%) had the lowest value but were not different from C2V pigs (21.09 ± 0.27%).

##### 3.1.3.5. Total protein

**Figure 7:**
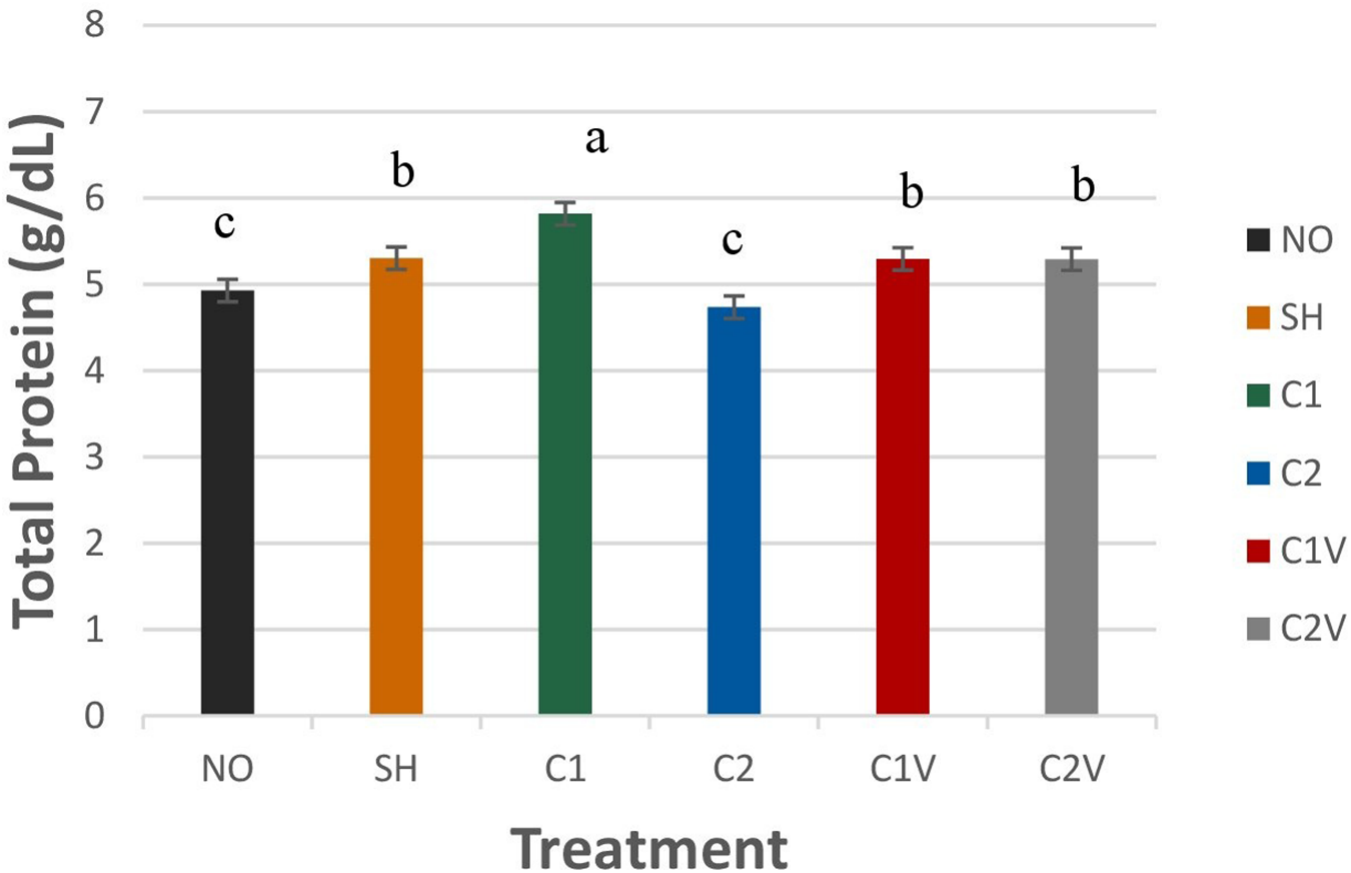
Study 1: LS means ± SEM for total protein (TP) for pigs castrated at 3 days of age using two methods of castration (1 incision, C1; 2 incisions, C2; or simulated castration, SH; or not castrated, NO) with or without the use of a vapocoolant (V) (*P* = 0.0001). *N* = 27 pigs per treatment group. ^a,b^Means with different superscripts differ at *P* < 0.05.

Total protein (TP) was significantly different among treatment groups (Figure 7; *P* = 0.0001). C2 treatment pigs (4.73 ± 0.13 g/dl) had a lower value than the other treatment pigs but were not different from the NO pigs (4.92 ± 0.13 g/dl). The C1 treatment group (5.82 ± 0.13 g/dl) had the highest TP levels compared to all other treatment groups (*P* < 0.05).

##### 3.1.3.6. Blood urea nitrogen

**Figure 8:**
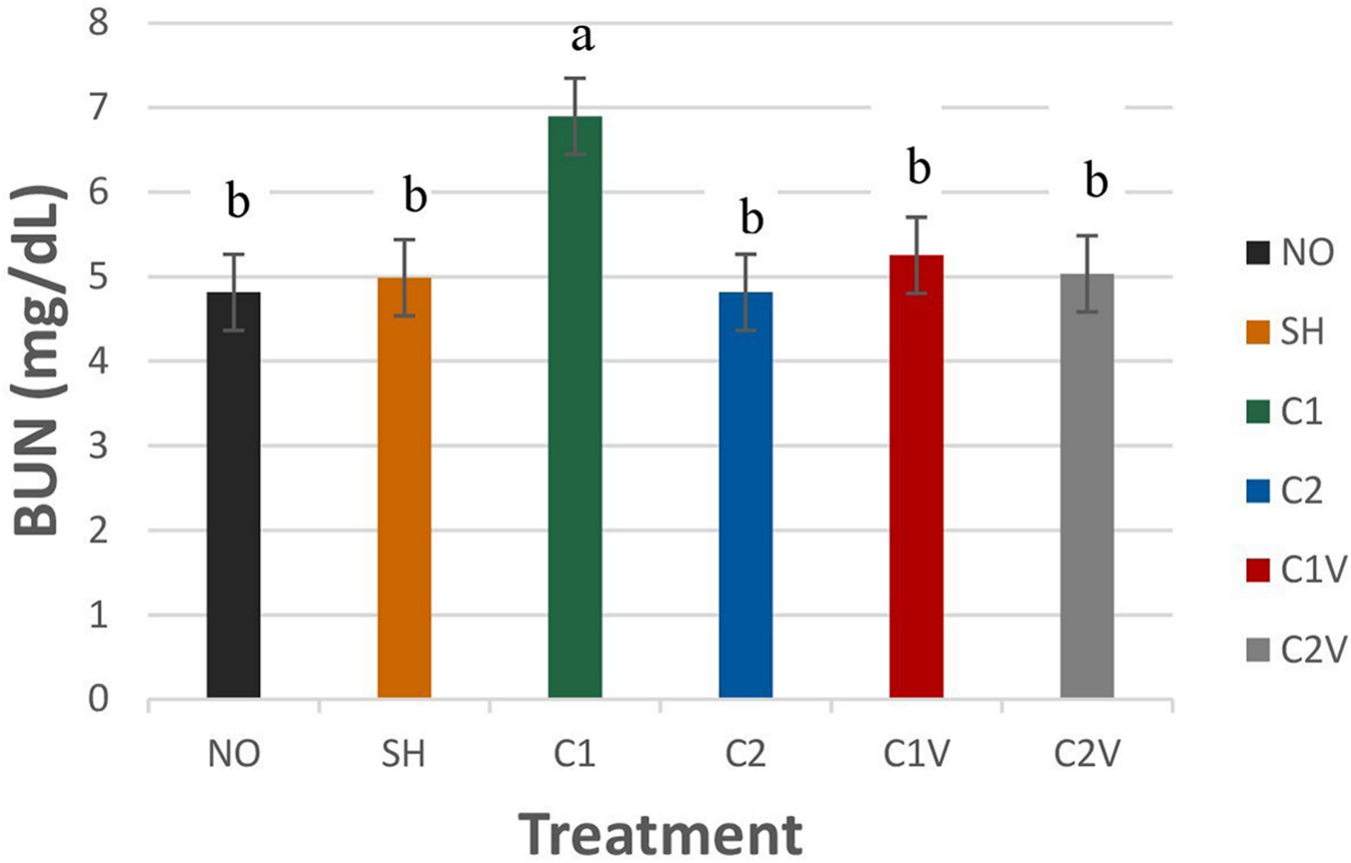
Study 1: LS means ± SEM for blood urea nitrogen (BUN) for pigs castrated at 3 days of age using two methods of castration (1 incision, C1; 2 incision, C2; or simulated castration, SH; or not castrated, NO) with or without the use of a vapocoolant (V) (*P* = 0.0413). *N* = 27 pigs per treatment group. ^a,b^Means with different superscripts differ at *P* < 0.05.

Blood urea nitrogen was significantly different among treatment groups (Figure 8; *P* = 0.0413). The C1 treatment group (6.89 ± 0.45 mg/dl) had the highest BUN value compared to all other groups (*P* < 0.05 mg/dl). There was no difference among the other treatment groups.

##### 3.1.3.7. Glucose

**Figure 9:**
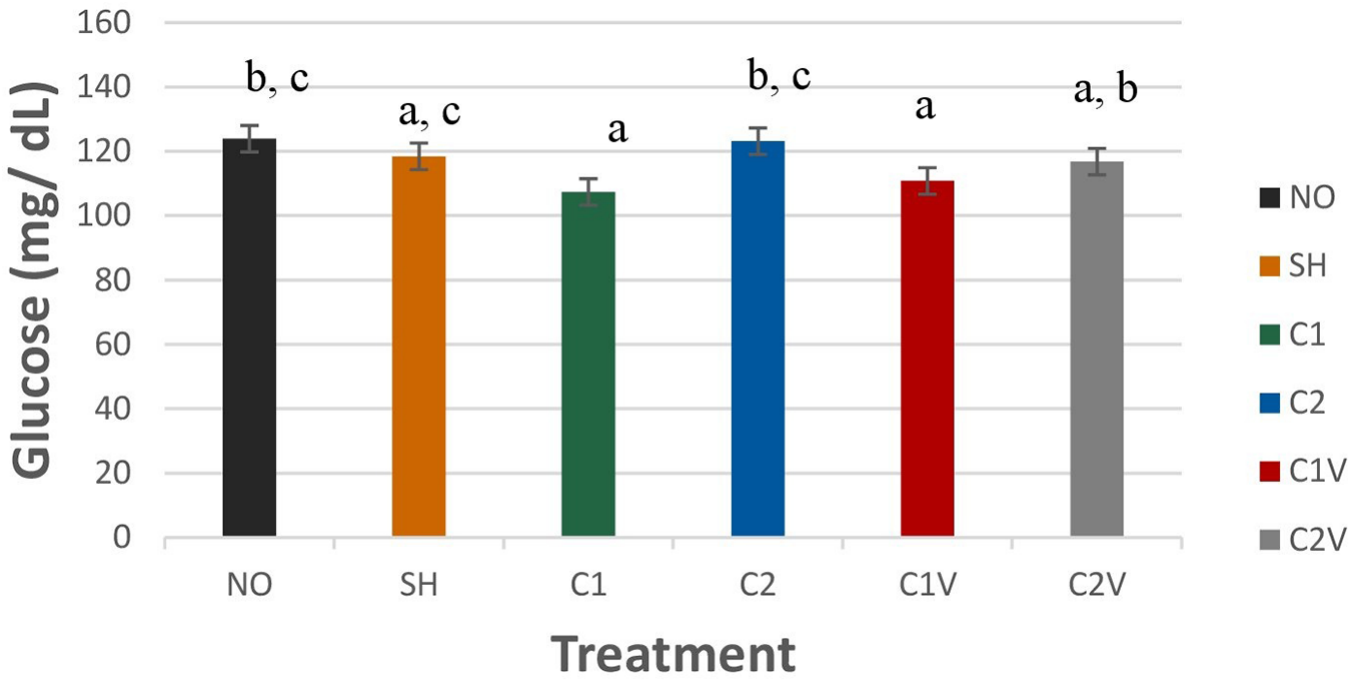
Study 1: LS means ± SEM for glucose (GLU) for pigs castrated at 3 days of age using two methods of castration (1 incision, C1; 2 incision, C2; or simulated castration, SH; or not castrated, NO) with or without the use of a vapocoolant (V) (*P* = 0.0223). *N* = 27 pigs per treatment group. ^a,b,c^Means with different superscripts differ at *P* < 0.05.

Glucose (GLU) was significantly different among treatment groups (Figure 9; *P* = 0.0223). GLU was highest for NO and C2 pigs (123.90 ± 4.10 and 123.14 ± 4.10 mg/dl, respectively). Although both groups were not significantly different from SH pigs (118.41 ± 4.10 mg/dl). C1, C1V, and C2V (107.35 ± 4.10, 110.79 ± 4.10, and 116.77 ± 4.10 mg/dl, respectively) were not significantly different from each other.

#### 3.1.4. Cortisol

Cortisol values were not significantly different among treatment groups.

#### 3.1.5. Wound scores

**Figure 10:**
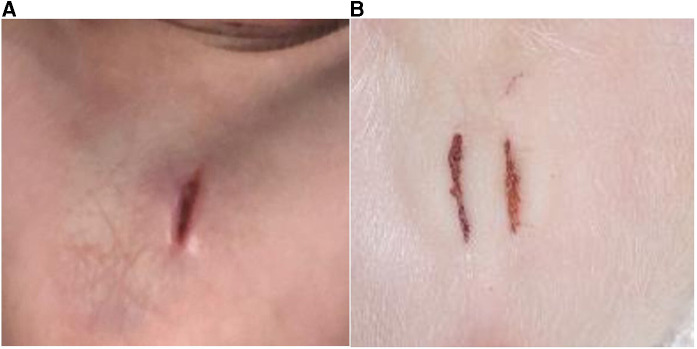
Shows the placement and size of incisions. (**A**): Single incision (C1); (**B**): double incision (C2).

Post-castration wound scores did not significantly differ among treatment groups. Figure 10 shows the placement and size of incisions.

### 3.2. Study 2

#### 3.2.1. Performance

Body weight and average daily gain (ADG) did not significantly differ among treatment groups at baseline, 24 h, 72 h, 7 days, or 14 days post-castration.

#### 3.2.2. Behavior

Pig behavior did not significantly differ among pigs, regardless of the treatment. Similarly, vocalizations among treatment groups were also not significantly different among treatment groups during castration (*P* = 0.94).

#### 3.2.3. Hematology and blood chemistry

**Figure 11:**
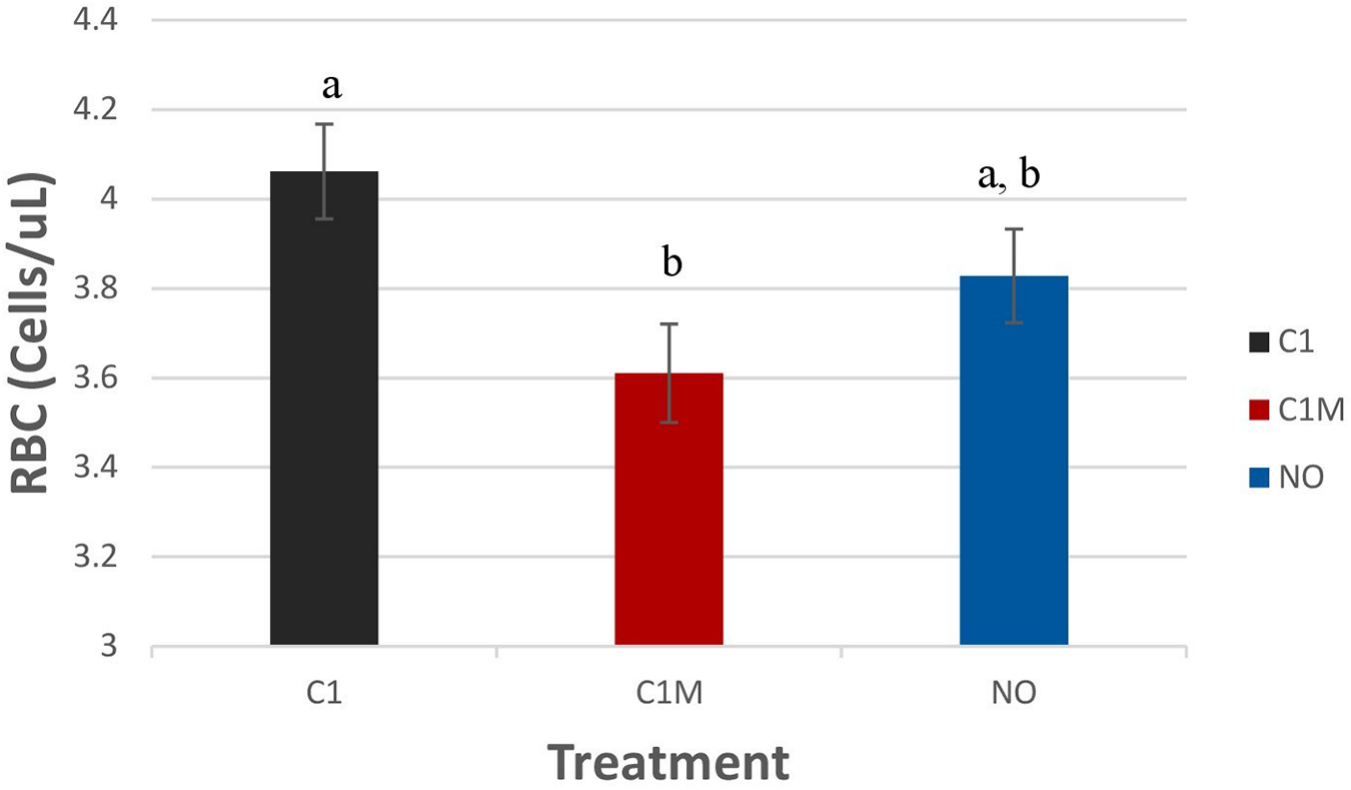
Study 2: LS means ± SEM for red blood cells (RBC) for pigs castrated at 3 days of age using two methods of castration (1 incision, C1; 1 incision plus Metacam®; C1M, or not castrated; NO) (*P* = 0.0304). *N* = 40 pigs per treatment group. ^a,b^Means with different superscripts differ at *P* < 0.05.

Blood measures were not significantly different among treatment groups, except for red blood cell numbers (*P* = 0.0304; Figure 11). Pigs in the C1 (4.06 ± 0.11 × 10^3^/µl) group had higher levels of RBCs compared to the C1M group (3.61 ± 0.11 × 10^3^/µl). However, C1 and C1M groups were not different from the NO treatment group (3.82 ± 0.10 × 10^3^/µl).

#### 3.2.4. Cortisol

**Figure 12:**
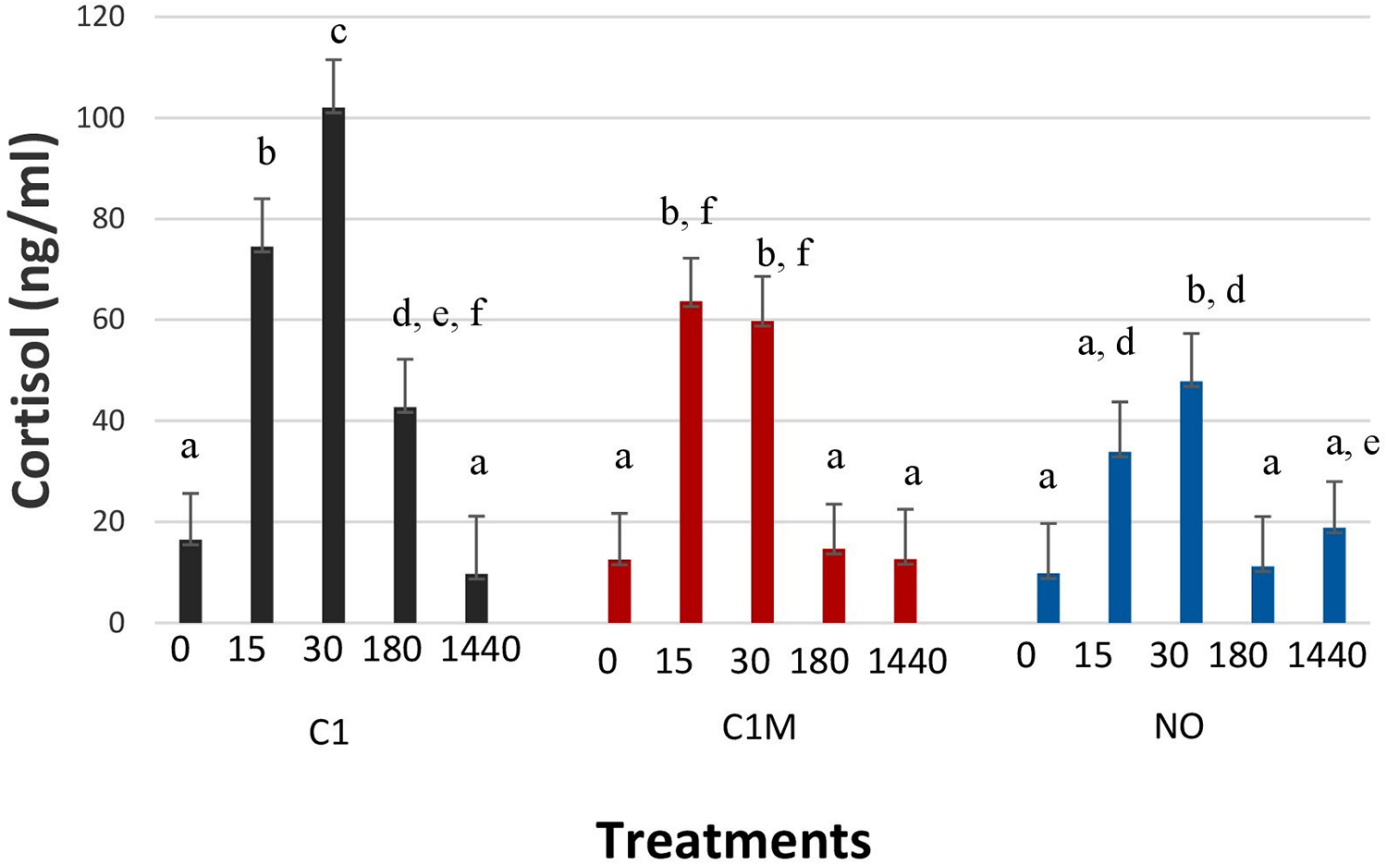
Study 2: LS means ± SEM for cortisol for pigs castrated at 3 days of age using two methods of castration (1 incision, C1; 1 incision plus Metacam®; or not castrated, NO). *N* = 40 pigs per treatment. ^a,b,c,d,e,f^Means with different superscripts differ at *P* < 0.05.

Cortisol values were significantly different among treatment groups over time (Figure 12; *P* = 0.047). At time 0 (baseline) all treatment groups had similar blood cortisol values. At 15 min post castration (T15) both C1 (74.48 ± 9.50 ng/ml) and C1M (63.65 ± 8.57 ng/ml) experienced an increase in cortisol concentrations significantly greater than the NO group (33.99 ± 9.89 ng/ml) but not different than each other. At T30, the C1 group (102.01 ± 9.5 ng/ml) had a greater cortisol concentration than both the C1M (59.76 ± 8.85 ng/ml), and NO group (47.81 ± 9.5 ng/ml). At T180, C1 (42.69 ± 9.50 ng/ml) had a higher cortisol concentration than C1M (14.65 ± 8.85 ng/ml), and the NO group (11.18 ± 9.90 ng/ml) but the C1M group was not significantly different than the NO group. At T1440 (24 h post-castration) there were no significant differences among treatment groups.

#### 3.2.5. Wound scores/bruising

Wound scores observed were within the range of 1–6 with a median score of 2 in the castrated pigs. Wound scores, specifically bruising, were not different between the C1 and C1M treatment groups.

## 4. Discussion and conclusion

Castration is a stressful and painful procedure for pigs (18, 34, 35). However, finding a method to reduce the pain of castration has proven to be challenging, as it can be time-consuming to apply and expensive. The main goal of this study was to identify if modification of the castration technique such as one incision versus two incisions would improve pig welfare, based on measures of performance, behavior, and physiology. An additional goal was to determine if analgesics, such as a topical ethyl chloride or an injectable analgesic, such as meloxicam, would mitigate the pain during or after castration or both.

Based on performance measures, pigs castrated with one incision (C1) and one incision plus ethyl chloride (C1V) had approximately 50 gm/day higher average daily gain (ADG) compared to the pigs castrated with two incisions with or without ethyl chloride (C2 or C2V). The 50 gm/day of higher average daily gain in one incision castration with or without ethyl chloride can be considered to have significant biological relevance compared to other two 2-incision castration groups pigs when a long production timeline is considered. The effect of C1 and C1V on performance measures indicated that these methods of physical castration were no different from non-castrated pigs. Though McGlone et al. (13) reported increased weight at weaning in piglets castrated at 14 days of age. Hay et al. (6) and Carroll et al. (34) found no significant effect of castration on the body weight of pigs than non-castrated pigs.

The behavioral data showed no differences among treatments in the hour post-castration nor during the 24 h period the pigs were observed except for vocalization behavior. This finding contrasts with other studies that have shown that there are behavioral changes after castration (6, 13, 18). All the pigs were handled multiple times after castration for weighing and blood sampling, which can be stressful and may have affected their behavior by masking some of the effects of castration among treatment groups. Vocalization data did show a difference among treatment groups. Pigs in the C1 and C1V treatment groups had louder scream but unlike C1, C1V did not differ from the C2 and C2V treatment groups. Pig scream, associated with the stress and pain induced during castration, should have a high sound power (dB) (36) and is different than other vocalization sound (19, 37). Von Borell et al. (37) reported 15 dB higher relative sound energy of vocalization compared to grunting and squealing sound. It was observed that it is more difficult to grasp the second testicle out of one incision versus two incisions. During castration when the incision was made on the skin and the first testicle was removed, there is a connective tissue called perineal raphe that needs to be incised in order to access the second testicle (38). When cutting through the perineal raphe, if the researcher cut too deep, the incision went all the way through the testicle. It was observed that incising through the testicle caused exaggerated pain to the pig, as a researcher recorded that the screams seemed louder during the training phase. The use of ethyl chloride seemed to mitigate some of the pain but not all as can be seen in the data where the use of the ethyl chloride during one-incision castration did not differ from two-incision castration and two-incision castration plus ethyl chloride. One-incision castration is not commonly used in the swine industry. Thus, if adopting this technique, the workers must be trained until proficient or the acute pain of cutting through a testicle is worse than the pain of 2-incision castration. Due to the shortage of labor in the swine industry, it will be difficult to adopt a technology that is more labor-intensive if the benefits are not economically significant.

Blood chemistry measures such as total protein (TP), blood urea nitrogen (BUN), and glucose (GLU) was found to be different between treatment groups. Total protein was higher in the C1 treatment group, which may indicate they were possibly not nursing as much, or they experienced more stress and protein catabolism. Total protein and albumin concentrations are markers for protein homeostasis, which increase with dehydration (39). However, reduced nursing behavior was not supported by the behavior data collected, as it showed no difference in nursing behavior among treatment groups. Additionally, the stress of handling and bleeding may have affected these values. This can be seen in the difference in TP values for NO and SH pigs. SH pigs were exposed to handling and weighing plus sham castration (which may have added more stress), possibly leading to the TP difference between NO and SH pigs. BUN values were also higher for the C1 treatment compared to the other treatment groups. Blood urea nitrogen is a metabolic waste product in the blood generated from the breakdown and probably due to muscle and protein breakdown caused by muscle exertion, and has been reported during transport (39). GLU was significantly different among treatment groups. GLU was the highest for NO and C2 pigs, but both groups were not significantly different from SH pigs. C1, C1V, and C2V were not significantly different than each other. There was no time effect. This may indicate that pigs continued to nurse during the 24 h post-castration. In this regard, E. Pérez-Pedraza et al. (38) found no significant differences in glucose concentration among treatment groups in a study on the effect of the number of castration incisions and the use of local anesthesia on physiological parameters. A difference in cortisol values among treatment groups was not identified. The cortisol findings support the use of meloxicam in reducing stress associated with castration at T30 and T180 post-castration. At T15, the stress of castration was high regardless of the use of meloxicam. This may indicate that the administration of the product was not effective at the time given and may need to be administered more than 15 min prior to castration.

Wound scores were also not different among treatment groups. This contrasts with Sutherland et al. (18) who used a topical anesthetic (lidocaine) to reduce the pain of castration and observed differences in wound healing at 9 and 14 days.

Based on measures of performance, behavior, and physiology it was determined that one incision castration would be used versus C1V because the differences were not great between the two treatments, and the data were not sufficiently clear to conclude that C1V was better than C1. C1V did not have a significant effect on reducing the pain of the incisions made during castration. In fact, when the castration incisions were made, pigs were not observed to squeal but mainly grunt. When the testicles were gripped and the spermatic cord was cut, pigs squeal at high frequencies and intensities. This is in agreement with other studies that have suggested that cutting the spermatic cord is more painful (17, 40, 41) significantly more than the incision (16). Thus, C1 was chosen to be part of Study 2.

Study 2 consisted of evaluating if one incision castration and the use of an anti-inflammatory 15 min prior to castration would reduce the pain of castration based on behavior, performance, and physiology. Performance measures did not differ among treatment groups. The behavioral data showed no difference among treatment groups in the hour post-castration nor during the rest of the 24 h period, the pigs were observed.

During surgical castration, the incision induces localized stinging pain of cutaneous origin, but the pain and vocalization are greater while extracting and severing the spermatic cord than sham handling or initial cutaneous incision (19). NSAIDs produce analgesia principally by suppressing the synthesis of prostaglandin and other inflammatory mediators during post-castration (42). NSAIDs may act to mitigate surgical stress and pain (42, 43) but the acute pain associated with scrotal incision and severing of the spermatic cord is not mitigated by NSAIDs like meloxicam (44). In a study, von Borell et al. (2) reported that 2 mg/kg BW meloxicam given intramuscularly 15 min before castration prevented a rise in cortisol concentration after castration, suggesting that it was effective in reducing pain. However, this study showed that the use of meloxicam was not effective at reducing pain, as vocalization data did not differ among treatment groups in this study. Consistent with this finding, Keita et al. (30) reported no significant effect of meloxicam in vocalizations for piglets surgically castrated without the administration of pre-emptive meloxicam. Similarly, Kluivers-Poodt et al. (40) using temporal, waveform, and spectral parameters to measure the vocal response during castration found no differences in vocalization in meloxicam-treated piglets compared to piglets surgically castrated without meloxicam.

Physiological measures included blood hematology and chemistry. Blood measures were not significantly different among treatment groups, except for red blood cells (*P* = 0.0304; Figure 10). C1 pigs had higher values than C1M pigs and No pigs. This may be due to a disruption in RBC synthesis, and blood clotting that may be impacted negatively by meloxicam. Cortisol values were different among treatment groups. The cortisol findings support the use of meloxicam in reducing stress associated with castration at T30 and T180 post-castration. At T15, the stress of castration was high regardless of the use of meloxicam. This may indicate that the administration of the product was not effective at the time given and may need to be administered more than 15 min prior to castration. In this regard, Keita et al. (30) found an increase in cortisol in blood for short time followed by a significant reduction post-castration in pigs that were administered meloxicam preoperatively only. Similarly, Zöls et al. (45) in a study on the effects of analgesics on the castration of piglets found significantly high cortisol concentrations in 1 and 4-h post-castration in piglets castrated without preoperative meloxicam whereas no significant rise in cortisol was noted in piglets administered with meloxicam preoperatively. Likewise, the wound score was not significantly different among the treatment groups.

This study had some limitations. First, a standard stainless-steel weight of 2 kg was used to calibrate the weighing scale and was used throughout the study to measure the weights of pigs. This could be a limitation as the linearity of the measurements was not established for weight greater than 2 kg.

In addition, it is not sure if meloxicam is an effective analgesic for acute pain of castration. Previous literature suggested it has some benefit in a castration model (46, 47). This is a general problem for farm animal analgesia products; few have been extensively tested in different species in different potentially painful situations. The marginal efficacy of meloxicam in this study suggests that meloxicam is not expected to modify the acute surgical pain but more to change postoperative pain associated with inflammation. In addition, we do not have a good indication of dose and timing for use of Meloxicam (and most analgesics) in farm animals experiencing different types of pain.

Likewise, Ethyl chloride was intended to address the acute pain associated with the procedure of castration. As with Meloxicam, ethyl chloride analgesic properties were not previously validated as effective in each painful experience.

In conclusion, the data collected gives sufficient insight into the benefits and limitations of one-incision castration compared to two-incision castration. The use of vapocoolant; ethyl chloride has no effect on reducing the pain associated with castration incisions. Additionally, meloxicam may have removed stress due to inflammatory pain, but no other effects were seen. Further research could potentially identify further positive and negative effects of meloxicam, and the correct timing and dose for administration to truly identify if it could mitigate the pain associated with one incision castration.

## Data Availability

The raw data supporting the conclusions of this article will be made available by the authors, without undue reservation.
